# Can probiotics benefit young people with autism spectrum disorders?

**DOI:** 10.1192/bjo.2021.752

**Published:** 2021-06-18

**Authors:** Pooja Ramani, Regina Sala

**Affiliations:** 1 Great Ormond Street Hospital; 2 East London NHS Foundation Trust

## Abstract

**Aims:**

The aims are to evaluate the effectiveness of Probiotics on young people with Autism Spectrum Disorder.

We hypothesized that there will be an improvement of the comorbid gastrointestinal symptoms that can accompany Autism Spectrum Disorder.

We believe that the use of probiotics can exert bidirectional effects on the gut-brain axis which may result in improvements in core Autism symptoms.

**Method:**

A literature search was performed in accordance with Preferred Reporting Items for Systematic Reviews and Meta-Analyses (PRISMA) guidelines. We used databases including OVID MEDLINE, Pubmed, EMBASE, AMED and the Cochrane register of controlled trials. Studies using Probiotics as a treatment for children with ASD were identified by key search terms; Child*, young person*, adoles*, teenagers, ASD, Autism Spectrum Disorder, Autism, Pervasive developmental disorder, PDD, Probiotics, Supplements, Lactobacillus, and Bifidobacterium. Inclusion criteria: Children of age range 2-18 with a diagnosis of ASD and having at least one gastrointestinal symptom were included. Exclusion criteria: The following were excluded: studies looking at Autism with interventions aside from Probiotics; studies where Probiotics were tested in conjunction with other interventions; studies where there were additional neurodevelopmental disorders.

**Result:**

Twelve studies identified all utilized probiotics. This included 7 Randomised Control Trials, 2 Open-Label studies, 1 pre and post-intervention design and 1 Case study. All RCTs gave probiotics or placebo to children.

Ten studies showed an improvement in gastrointestinal symptoms. Six studies showed improvements in various behavioral measures. Four studies showed improvements in core autism symptoms. However, the sample sizes in these studies were not large enough to prove statistical significance.

**Conclusion:**

No studies showed an adverse reaction which indicates probiotics can be considered a safe treatment.

The improvements in a variety of parameters imply probiotics a suitable adjunctive intervention that may help improve ASD core symptoms in young people as well as improving physical and behavioural comorbidities which in some cases was noted by parents.

However, due to high dropout rates and generally small sample sizes, larger-scale trials are needed to critically confirm the efficacy of probiotics for children with ASD.

